# Photoacids and Photobases: Applications in Functional Dynamic Systems

**DOI:** 10.1002/anie.202422963

**Published:** 2025-01-31

**Authors:** Anna Yucknovsky, Nadav Amdursky

**Affiliations:** ^1^ Schulich Faculty of Chemistry Technion – Israel Institute of Technology Haifa 3200003 Israel; ^2^ Chemistry Research Laboratory University of Oxford 12 Mansfield Road Oxford OX1 3TA; ^3^ Chemistry School of Mathematical and Physical Sciences University of Sheffield Sheffield S3 7HF United Kingdom

## Abstract

Brønsted photoacids and photobases are a unique class of molecules that undergo a major change in their p*K*_a_ values between their ground and excited states, resulting in donating or accepting a proton, respectively, but only after light excitation. This property of photoacids/photobases makes them an attractive tool for light‐gating various dynamic processes. Here, we review the use of this property to manipulate functional dynamic systems with light. We discuss how a proton transfer event that can happen upon light excitation from a photoacid to a chemical moiety of a certain system or, vice versa, from the system to a photobase, can result in a shift in the equilibrium of the system, resulting in some dynamicity. We detail various systems, including self‐assembly processes of nanostructures, self‐propulsion of droplets, catalysis for hydrogen evolution or CO_2_ capturing, nanotechnological devices based on enzymatic processes, and changes in proton‐conducting ionophores and materials. We detail the basic guidelines for using Brønsted photoacids and photobases in a desired system and conclude with the current technological gaps in further using these molecules.

## Graphical abstract

Brønsted Photoacids and Photobases represent a unique class capable of donating or accepting a proton only after excitation. Here, we review the utilization of such molecules to light‐trigger the direction and amplitude of functional dynamic systems. We discuss the guidelines of how to use such molecules in various systems and give a futuristic outlook on the current technology gap for further utilization.

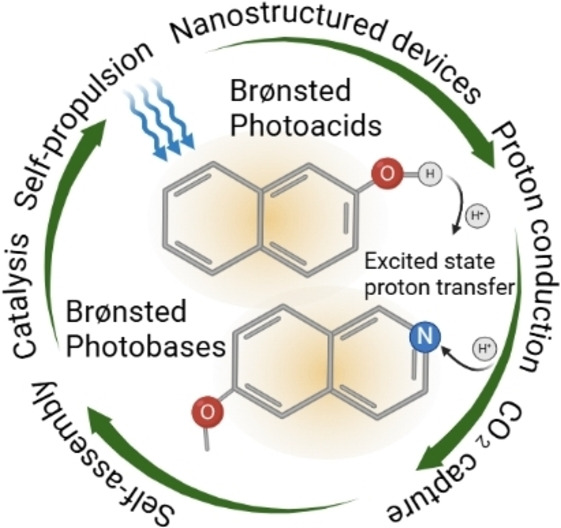

## 1 Introduction

**Figure 1:**
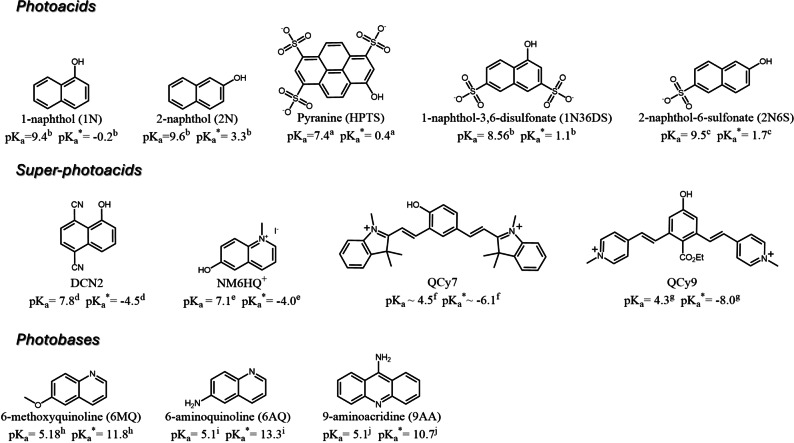
Common Brønsted photoacids and photobases and their p*K*_a_ values. The values are taken from a. Ref.,^[37]^ b. Ref.,^[1]^ c. Ref.,^[38]^ d. Ref.,^[39]^ e. Ref., f. Ref.,^[40]^ g. Ref.,^[41]^ h. Ref.,^[42]^ i. Ref.^[43]^ j. Ref.^[44]^

This review will cover recent advances in using light as a gating source to control dynamic reactions using photoacids and photobases and give guidelines for their use while designing new dynamic systems. There is considerable ambiguity in the use of the terms photoacids and photobases. Hence, we first need to distinguish between the different classes (Table 1). One of the earliest applications of molecules that are termed photoacids is of molecules that act as photoacid generators. These molecules undergo an *irreversible* light‐induced photolysis, resulting in the formation of an acid. The resultant acid can be used in various reactions, such as in polymerization, crosslinking, or photolithography. We will not discuss this class in this review. The second class of photoacids are molecules that undergo a *reversible* light‐induced structural change (ring opening/closure), whereas the spiropyran‐merocyanine system is the most common representative of this class. Due to the involvement of substantial structural change in this latter class, the reversibility process can be slow (seconds to hours). We will also not discuss this class in this review. Unlike photoacids, photobases are much less used, but also here, we should distinguish between different classes. The first class is termed Arrhenius photobases, which are molecules that undergo a *reversible* light‐induced OH^‐^ dissociation, which is also a slow process. We will not discuss this class in this review. Here, we will review only molecules that demonstrate a dramatic change in the acidity and the basicity happening in their electronically excited state and, accordingly, can be termed as Brønsted type. For photoacids, such molecules are hydroxyarils (ROH) with a considerably lower p*K*_a_* in the excited states than the p*K*_a_ in the ground state (Figure 1).[^1^, ^2^, ^3^, ^4^, ^5^, ^6^, ^7^, ^8^, ^9^, ^10^, ^11^] The most common photoacids are pyranine (HPTS), naphthols, and hydroxycoumarins. A sub‐class of photoacids is termed ‘super photoacids’ with very low p*K*_a_* values of < −2, such as some naphthol or cyanine derivatives (Figure 1).[^12^, ^13^, ^14^, ^15^, ^16^, ^17^, ^18^, ^19^, ^20^, ^21^, ^22^, ^23^, ^24^, ^25^, ^26^] For photobases, such molecules commonly have a nitrogen heteroaromatic (RN), with a considerably higher p*K*_a_* in the excited state than the p*K*_a_ in the ground state (Figure 1).[^10^, ^27^, ^28^, ^29^, ^30^, ^31^, ^32^, ^33^, ^34^, ^35^, ^36^] The most common photobases are aminoquinolines and aminoacridines. Brønsted photoacids/photobases undergo a reversible light‐induced excited state proton transfer (ESPT) process. However, unlike the previously mentioned classes, the reversible process is very fast (nanoseconds to microseconds). As will be detailed in this review, the mechanism, reversibility, and timescales of the ESPT process distinguish Brønsted photoacids/photobases from all other classes. *The major difference is that while the other classes result in a stable pH jump of an aqueous solution, Brønsted photoacids/photobases do not result in that*. Accordingly, it also dictates the type of applications that can be gated using this class.

**Table 1:** Different classes of photoacids and photobases.

Photoacids	Definition	Photobases	Definition
Brønsted	Photoacids that release a proton in their excited state. Fast reversible process in the ground state	Brønsted	Photobases that capture a proton in their excited state. Fast reversible process in the ground state
Photoswitches	Photoacids that release a proton following photoisomerization. Slow reversibility with respect to Brønsted photoacids	Arrhenius	Photobases that release hydroxide ions in their excited state. Slow reversibility with respect to Brønsted photobases
Photoacid generators	Irreversible photoacids. Molecular cleavage	Photobase generators	Irreversible photobases. Molecular cleavage

### 1.1 A Brief History of Brønsted Photoacids/Photobases

**Figure 2:**
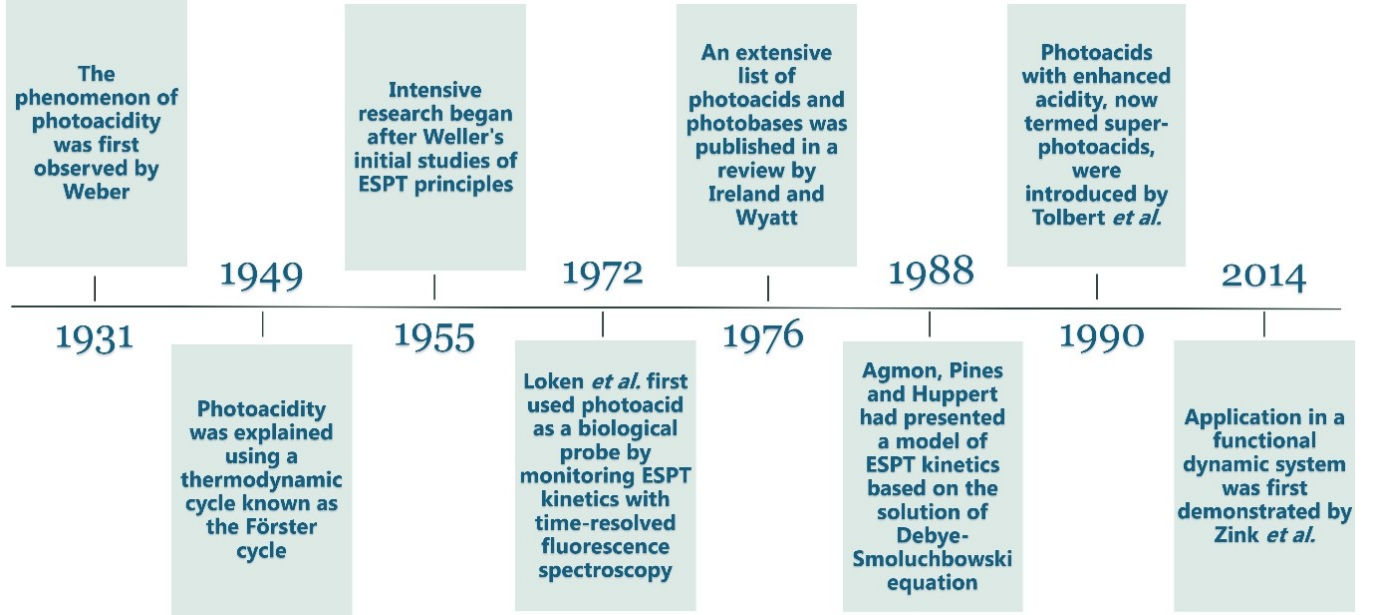
The timeline of the photoacid and photobase research.[^10^, ^45^, ^49^, ^50^, ^51^, ^52^]

The research on Brønsted photoacids/photobases emerged almost a hundred years ago (Figure 2). Weber was the first to observe a p*K*_a_ shift in some organic molecules in their electronically excited state, followed by Förster's explanation of the phenomenon by suggesting a thermodynamic cycle to estimate the excited state p*K*_a_*, known as the Förster cycle, which was further elaborated by Weller.[^10^, ^45^, ^46^, ^47^, ^48^] The idea of using photoacids as biological probes emerged in the late 1960s.^[45]^ A few years later, Loken et al. showed that the ESPT process can be used to study the environment of a photoacid probe molecule.^[49]^ With the advance of time‐resolved spectroscopy, using photoacids as ESPT probes has become popular, with the pyranine (HPTS) probe being the leading photoacid used for such applications.^[37]^ With time, more Brønsted photoacids/photobases have been introduced. From a theoretical perspective, the kinetic model based on a diffusion process of the proton following dissociation from a photoacid was introduced in the 1980’s.^[50]^ Later, Tolbert et al. studied the relationship between the structure of photoacids and their strength, which led to the development of super‐photoacids with very low p*K*_a_* values.^[51]^ Only in the recent decade, new applications of photoacids and photobases have emerged in functional dynamic systems, which are based on light‐induced transient proton release or uptake for reversibly controlling various processes.^[52]^

### 1.2 Theoretical Aspects of Photoacidity and Photobasisity

**Figure 3:**
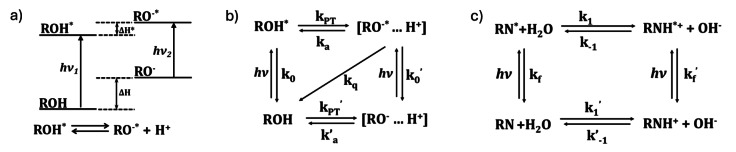
**a**) Förster cycle of a photoacid. ΔH and ΔH* indicate the changes in the enthalpy in the ground and excited states, respectively; h is a Plank's constant, ν_1_ and ν_2_ are the frequencies of S_0_ to S_1_ (0–0) transitions of the protonated (ROH) and deprotonated (RŌ) photoacid, respectively. Interception points between the normalized absorption and emission spectra of ROH and RŌ indicate 0–0 transitions.[^5^, ^53^] **b**) Photoprotolytic cycle of a photoacid.^[10]^ **c**) Proton abstraction cycle of a photobase.^[29]^

The Förster cycle graphically presents the thermodynamic equilibrium of the photoacid dissociation in the ground and excited states (Figure 3a), which is based on a different energy transition (hν) of the protonated and deprotonated forms. From the Förster cycle, assuming that ΔH* ‐ ΔH ≈ ΔG^0^* ‐ ΔG^0^, one obtains:
(1)

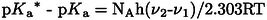




where *N*_A_ is Avogadro's number, h is Plank's constant, R is the gas constant, and T is temperature. The same equation can be used for photobases; in this case, the absorption band frequency of the deprotonated state is higher than that of the protonated state, resulting in a positive value for ν_2_‐ν_1_ in eq 1. It's important to note that to derive eq. 1 from the Förster cycle, certain assumptions must be made: (1) the ΔH and ΔH* are the same as in the standard state; (2) protonation entropy must be the same in the ground and excited states, and (3) the frequencies of 0–0 transitions are known.^[10]^ From an experimental point of view, the ground state p*K*_a_ is easy to determine using UV/Vis absorption and pH titration because of the different absorption bands of the protonated and deprotonated forms. The p*K*_a_* is commonly determined using the Förster cycle. Our first good practice tip here is to use both absorption and emission to calculate the difference in energy transitions between the forms.

The mechanism of ESPT is usually described by the photoprotolytic process (Figure 3b and **3 c** for photoacids and photobases, respectively). Upon light excitation, a very fast (attoseconds) charge redistribution is happening, resulting in a rearrangement of the electron density away from the OH group or toward the N group and, subsequently, rearranging the H‐bonds, which happens within femtoseconds. This step also determines whether a photoacid is ‘regular’ or ‘super’, whereas super photoacids have a more substantial electron‐withdrawing effect or resonancy in the excited state.[^12^, ^13^, ^14^, ^15^, ^16^, ^39^, ^40^, ^53^, ^54^, ^55^, ^56^, ^57^, ^58^] The next step is the ESPT process. At this stage, it is of prime importance to mention that, *as with any other Brønsted acid/base, there is a need for a proton donor or acceptor for the process to happen*. The ESPT process involves proton dissociation/association, solvation, and mobility (on the sub‐ns timescales). For photoacids, the released proton can reversibly geminate recombine with the excited conjugate base (RŌ) or interact with it in a quenching process (within 100 ps to 100 ns). For photobases, the re‐dissociation in the excited state is not explored very much. Eventually, following the return to the ground state, the conjugate base/acid rebinds the proton or re‐dissociates on the ns to ms timescale.^[2]^

The strength of a photoacid, i.e., how low the p*K*_a_* is, dictates the kinetics of the ESPT process, whereas usually, the lower the p*K*_a_*, the faster the process (for photobases, it is less explored).^[17]^ For strong super photoacids, the kinetics can be limited even by solvent orientational motion or vibration of the hydrogen bond between the photoacid OH group and the solvent.^[17]^ From an applicative perspective, *the* p*K*_a_ * dictates which proton acceptor can be used for the photoacid or which proton donor can be used for the photobase. For photoacids, while referring to the solvent as the proton acceptor, ‘regular’ photoacids, such as HPTS, can donate their proton only to water, while stronger photoacids can donate their proton to other protic solvents, such as methanol or ethanol. However, as stated, the ESPT to/from a photoacid/photobase and the solution does not change the pH of the solution in a stable manner due to the rapid re‐association/dissociation in the ground state. Hence, for using Brønsted photoacids/photobases in light‐gated dynamic systems, there is a need for a direct or solvent‐mediated ESPT process between the photoacid/photobase and the proton acceptor/donor in the functional system.

## 2 Applications in Functional Dynamic Systems

A system is considered dynamic when it is out‐of‐thermodynamic equilibrium. Complex out‐of‐equilibrium systems characteristic of living organisms have inspired the development of artificial ones capable of performing various functions such as self‐assembly, motion, signalling, information processing, and more.[^59^, ^60^, ^61^, ^62^, ^63^] Each system requires an energy source to maintain its function. Light is a form of energy that can be delivered remotely to a precise location without generating waste and can be converted into a chemical stimulus by molecules that undergo chemical changes upon excitation. For example, photoisomerization processes (e.g., for azobenzene) can impart a different geometry, dipole moment, and electronic structure that can influence surrounding molecules in the system and regulate processes, thus making the entire system responsive to the stimuli, i.e. dynamic.[^64^, ^65^]

*A general guideline for using Brønsted photoacids and photobases in dynamic systems*: Photoacids and photobases can mediate light control over pH‐sensitive processes such as acid‐base, redox, photoelectrochemical reactions or electrostatic interactions through the ESPT mechanism. However, as stated, Brønsted photoacids and photobases do not result in a stable pH change of the solution, and there is a need for a direct or solvent‐mediated ESPT from the photoacid and a proton acceptor or from a proton donor to the photobase. Since any PT process involving acid/base equilibrium is dictated by the ΔpK_a_ between the proton donor and the proton acceptor, the chemical moiety that is accepting/donating the proton from/to the photoacid/photobase should have a p*K*_a_ value in between the ground state p*K*_a_ and the excited state p*K*_a_* of the photoacid/photobase. Taking into consideration the values of common photoacids and photobases (Figure 1), the most common chemical moiety to target is carboxylic acid/carboxylate. Super photoacids can also donate a proton to highly acidic proton acceptors. Below, we will review several dynamics systems.

### 2.1 Self‐Assembly/Association

**Figure 4:**
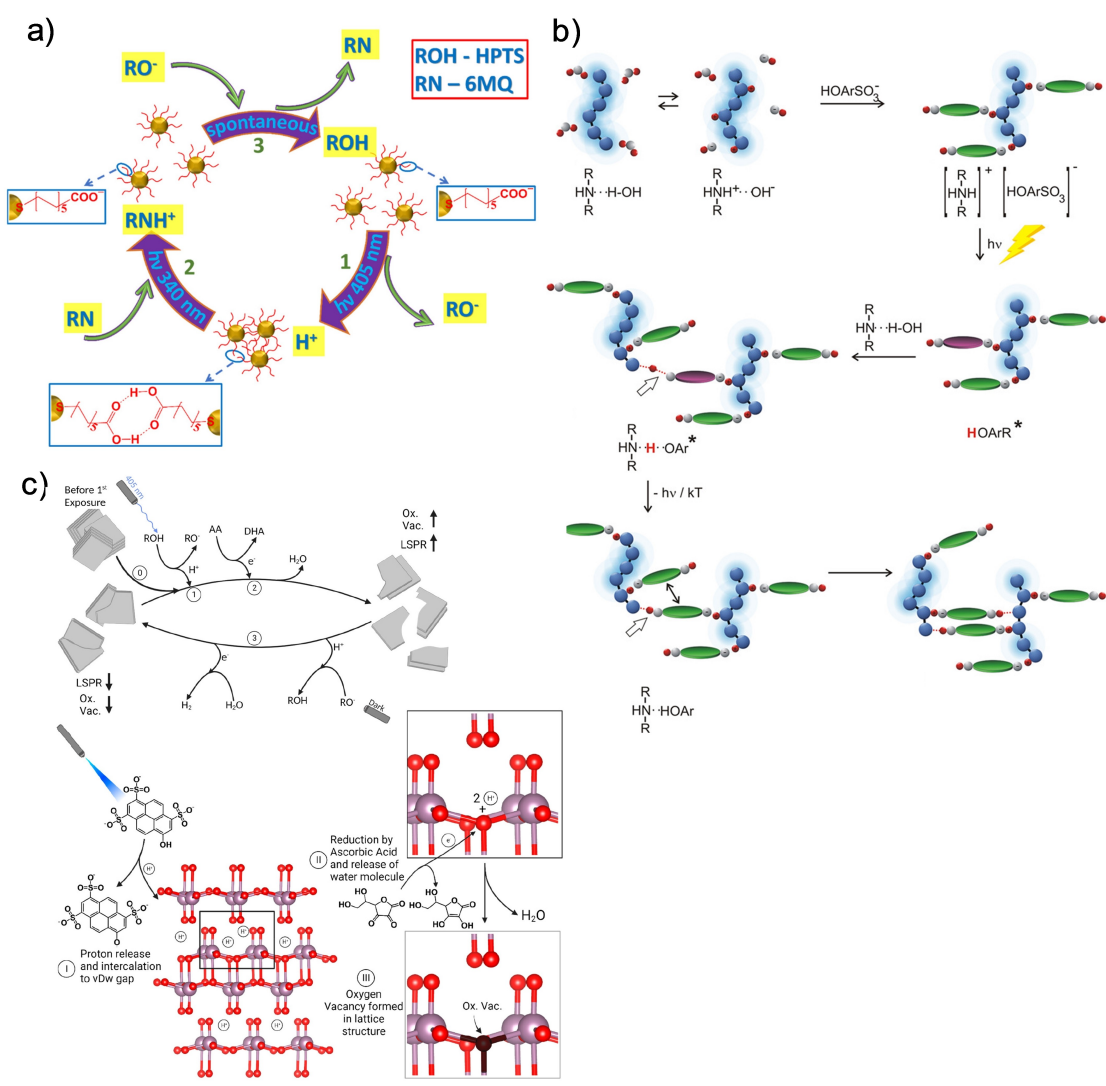
Light‐induced self‐assembly. **a**) Dynamic self‐assembly of gold nanoparticles decorated with ligands capable of reversible protonation. Upon the excitation of the photoacid, the ligands undergo protonation, resulting in particle aggregation. Upon the subsequent excitation of the photobase, they undergo deprotonation, and the aggregates disassemble. Reproduced with permission from Ref.^[42]^ **b**) Polyethylene imine polybase and a photoacid are structural units that create a network of hydrogen bonds. The process is initiated upon the excitation of a photoacid and leads to the formation of supramolecular structures. Reproduced with permission from Ref.^[66]^ **c**) Molybdenum oxide nanosheets consist of layers connected by van der Waals forces. Upon the intercalation of protons released by photoacids between these layers, a series of processes are initiated, resulting in the nanosheet breakage. In the dark, the nanosheets reassemble. Reproduced with permission from Ref.^[70]^

The first system we review is the association/dissociation of a chemical moiety following protonation. As stated, carboxylates can accept a proton from photoacids. In this way, any particles decorated with carboxylated ligands are electrostatically repelled due to the negative charge. However, following a direct ESPT process from a photoacid in the solution to the ligands, they become carboxylic acid, which allows the association between the particles through hydrogen bonds. In an opposite manner, a photobase can accept a proton from the carboxylic acid, thereby resulting in their re‐dispersion following excitation. In one of the explored systems, photoacids (HPTS) and photobases (6MQ) were alternately illuminated (with 405 and 340 nm light sources, respectively, for 10–20 seconds). During these illumination cycles, nanoparticles underwent reversible self‐assembly (Figure 4a).^[42]^ Other examples include the association of polymers, which was initiated by photoacids by forming hydrogen bonds (Figure 4b).[^66^, ^67^, ^68^, ^69^] In the system presented in Figure 4b, a photoacid (1‐naphthol‐4‐sulfonate) was a building block in the nano assembly. The second building block, polyethylene imine polybase, had both protonated and non‐protonated amine groups, which could form non‐covalent bonds with the sulfonate and hydroxyl groups of the photoacid in its ground and excited states, respectively. ESPT processes between the photoacid and non‐protonated amine groups of the polybase resulted in stable hydrogen bonds in the ground state. Hence, the associated structures could not spontaneously return to their original state, i.e., they were irreversible. Similar to this, nano‐assemblies of photoacids and poly‐cationic proton acceptors, e.g., dendrimers, were either irreversible[^66^, ^67^, ^68^] or reversible upon the addition of a regular base.^[69]^ In the latter system, nanostructures containing 1 N3,6DS photoacids and poly(amidoamine) dendrimers were triggered to reversibly form structures of different shapes and sizes via a ‘waste‐generating’ chemical stimulation.

Another mechanism for utilizing ESPT for self‐assembly/association processes is via ESPT‐induced redox processes. An example of it is a system involving molybdenum oxide nanosheets, dissolved in a solution containing a HPTS photoacid. In that system, the irradiation (405 nm) resulted in an ESPT process from the photoacid to the nanosheets and the intercalation of protons between the layers of the latter. In turn, a reducing agent present in the solution induced a redox process that resulted in the generation of oxygen vacancies in the atom array of the nanosheets, followed by their breakage(Figure 4c).^[70]^ Upon turning off irradiation, the nanosheets underwent oxidative healing, i.e., refilling of the oxygen vacancies, thus promoting their re‐association.

### 2.2 Self‐Propulsion

**Figure 5:**
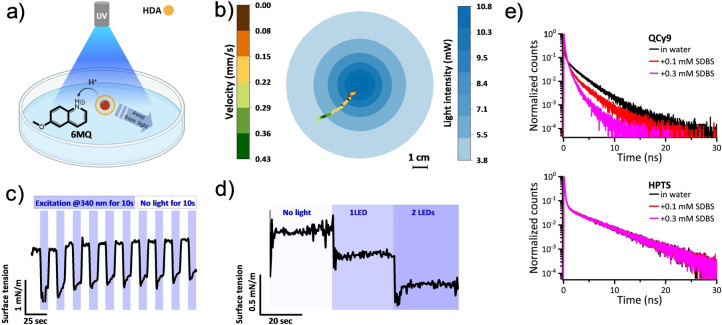
Self‐propulsion of oil droplets. **a**) schematic representation of a typical experimental setup demonstrating proton transfer from the droplet surface to the photobase with asymmetric light intensity in the middle and at the edge of the Petri dish. **b**) Processed and plotted data collected with a digital camera while monitoring the self‐propulsion. **c**) and **d**) The effect of light on the ST. Reproduced with permission from Ref.^[38]^ **e**) Comparison between the fluorescence decay of super photoacid QCy9 and regular photoacid HPTS in the presence of sodium dodecylbenzenesulfonate (SDBS). In the upper Figure, the shorter lifetime of the QCy9 fluorescence upon the addition of SDBS is due to the scavenging of the protons by the latter. This is not the case for HPTS, as it cannot protonate SDBS. Reproduced with permission from Ref.^[41]^

If a titratable moiety is present on the surface of a floating microscopic element, a proton transfer event to/from the moiety will change the surface charge and, accordingly, the surface tension. If the change is unevenly distributed along the surface, the breaking of the surface tension symmetry results in internal convective flows (a.k.a. the Marangoni effect), which might lead to a propulsion movement. In this way, photoacids and photobases were used to self‐propel oil droplets following an ESPT process from/to photoacids/photobases and titratable carboxylated groups on the droplet‘s surface (Figure 5a–**d**).^[38]^ To allow the symmetry breakage in surface tension, the droplets were irradiated gradiently using an LED or a laser light source in the presence of either photobases (6MQ) or photoacids (2 N6S or HPTS) in solution. Hence, one side of the droplet exhibited a different degree of ESPT processes than the other one. Subsequently, the droplet can self‐propel either toward or away from the light. The droplet self‐propulsion system was also used to demonstrate one of the few applications of super phototocids. As stated, unlike regular photoacids, super photoacids can induce an ESPT process to highly acidic groups. This can be followed with time‐resolved spectroscopy techniques, e.g., time‐correlated single photon counting (TCSPC). Figure 5e shows the fluorescence decay of the super photoacid QCy9 (the protonated form) in the presence of a weak (acidic) sulfonate‐based proton acceptor (SDBS) in comparison to the decay of the regular photoacid HPTS under the same conditions. For QCy9, the decay is faster with the addition of SDBS since an ESPT process is possible between QCy9 and SDBS. Unlike QCy9, HPTS cannot undergo ESPT to SDBS, hence, its fluorescence decay is not affected by the latter. In the described system, the QCy9 super photoacid was used to protonate SDBS on the droplet‘s surface, resulting in self‐propulsion.^[41]^

### 2.3 Nanostructured Devices

**Figure 6:**
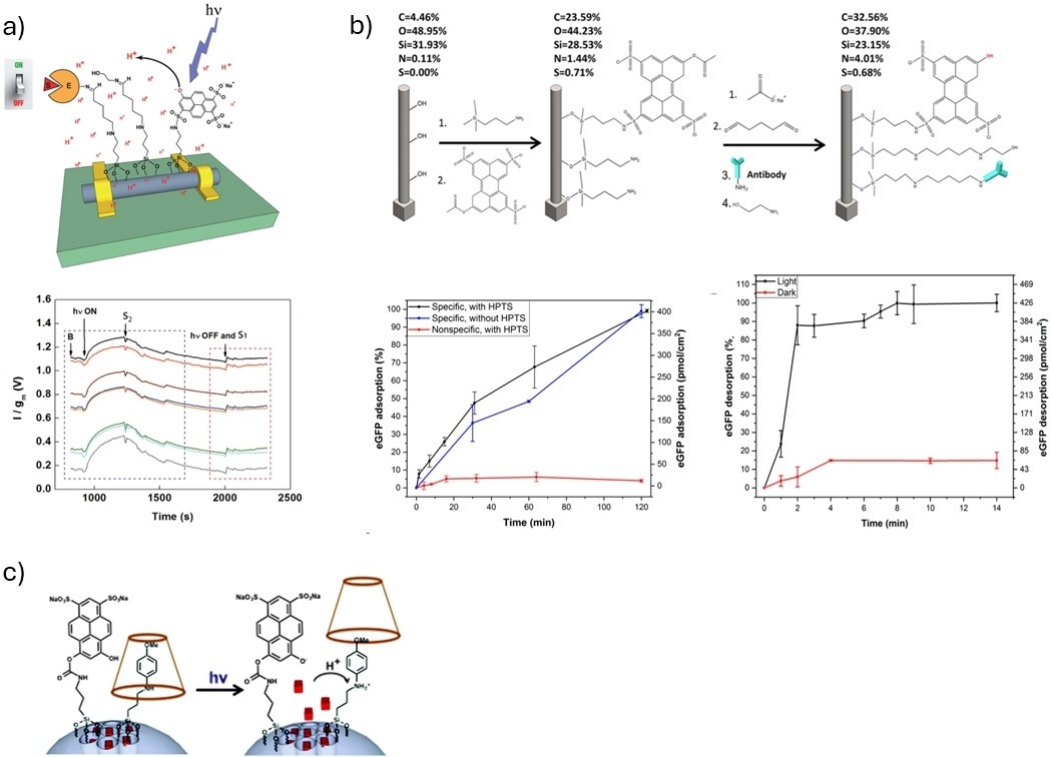
Nanostructured devices. **a**) Top: the schematic representation of HPTS‐enzyme‐SiNW FET, bottom: pepsin enzymatic activity triggered by light, *n*=8 devices, S= the substrate, g_m_ is transconductance, and I is current. Reproduced with permission from Ref.^[71]^ **b**) Top: schematic representation of the photoacid and antibodies immobilization on the nanopillar surface, bottom: adsorption and desorption of the model GFP protein as a function of time. Reproduced with permission from Ref.^[72]^ **c**) Schematic representation of light‐activated nanovalve. Reproduced with permission from Ref.^[52]^

Until now, we have primarily discussed an ESPT process of soluble photoacids/photobases to/from a certain acceptor/donor. While this approach works, it is a wasteful approach as only the excited photoacids/photobases that are close to the proton acceptor/donor are participating in mediating the dynamic process, while the vast majority of excited molecules in the solution will not participate in the process. Accordingly, some approaches have confined the location of the photoacid. Here, we will discuss the confinement within a nanostructured device. In an example where a silicon nanowire field‐effect transistor (SiNW FET) was used, the HPTS photoacid was bound on the SiNW (Figure 6a).^[71]^ In parallel, an additional pH‐responsive element was also bound on the SiNW. This element was an enzyme (pepsin), which is more active at low pH values, and the activity of the enzyme can be followed using the FET configuration. Accordingly, upon irradiation with 405 nm light, the ESPT from the photoacid to the solvent in a very close vicinity to the enzyme was shown to influence (increase) its activity (Figure 6a). In a conceptually similar manner, HPTS was bound on silicon nanopillars in close proximity to antibodies (Figure 6b).^[72]^ The antibody‐antigen association is pH dependent, whereas the stronger binding is around neutral pH. In this way, the light‐triggered ESPT from the photoacid to the solvent near the antibody‐antigen complex can result in its dissociation. This system was further developed in subsequent studies by fabricating branched pillars with increased surface area^[73]^ and decorating them with DNA aptamers.^[74]^ One of the advantages of using nanopillar‐decorated surfaces is their significantly large binding area, which enables the use of such devices for selective separation and pre‐concentration of molecules in complex samples. The last example of the use of a nanomaterial is nanovalves, on which the HPTS photoacid was bound next to a small molecule that can bind the cargo molecule (in this study, it was propidium iodide).^[52]^ Since the latter binding is pH‐dependent, the ESPT from HPTS (with a 408 nm pump later on the minutes‐to‐hours time scales) has resulted in the protonation of the molecule holding the cargo, and the dissociation of the cargo from the surface of the silica nanoparticle (Figure 6c)

### 2.4 Catalysis

**Figure 7:**
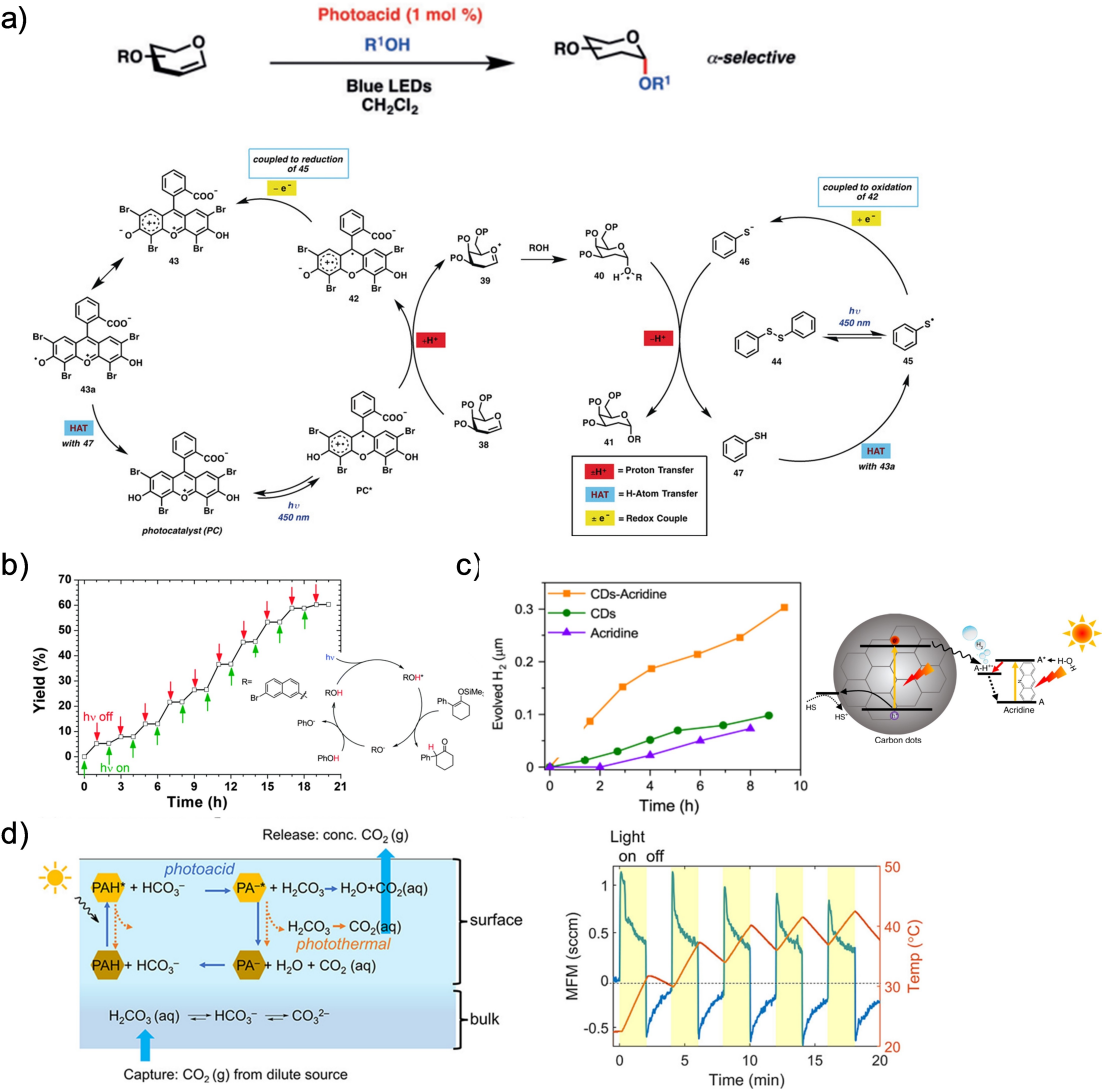
Catalytic systems. **a**) The stereoselective conversion of glucans to 2‐deoxyglycosides and the suggested catalytic cycle. Reproduced with permission from Ref.^[75]^ **b**) The suggested photocatalytic protonation cycle of 1‐phenyl‐2‐(trimethylsiloxy) cyclohexene upon light excitation (367 nm) of 7‐bromo‐2‐naphthol photoacid and the effect of light on the reaction yield. Reproduced with permission from Ref.^[76]^ **c**) Schematic representation of the mechanism and measurements of the hydrogen evolution. Reproduced with permission from Ref.^[78]^ **d**) The working principle of the photochemical CO_2_ capture and release and the effect of light on the gas flow (MFM).^[79]^

From an applicative point of view, one of the most promising uses of photoacids and photobases to control dynamic reactions is in the field of (photo−)catalysis. Here, the ESPT process can participate in a protonation catalytic process as a reversible light‐triggered source of proton (for photoacids) or a proton acceptor (for photobases). This concept can be used even in organic synthesis (Figure 7a and b).[^75^, ^76^] Figure 7a displays a rationally designed acridinium‐based phenol‐substituted organic photoacid, which was used to activate (with blue light) synthetic glycals to produce 2‐deoxyglycosides. In the absence of molecules in solution that can act as proton donors/acceptors, two catalytic reactions in a single photoacid cycle can be envisioned: one is the ESPT process for the primary catalytic reaction, while the other is the ground state recombination process, thus taking a proton from another molecule. Similar concepts for using photoacids in catalysis have also been introduced to glycosylation, acetalization, and arylation processes.^[77]^

As the ESPT process involves transferring a proton, one of the immediate photo‐catalytic processes that can benefit from it is H_2_ generation. A hydrogen generation process involves electron and hole pair generation, separation, and transfer, which is usually coupled with a proton transfer. The excited electron transfer to the proton is suggested to be a rate‐limiting step. Accordingly, the ultrafast ESPT process of Brønsted photoacids/photobases is highly advantageous here. In one of the examples of this process, a photobase (acridine) was incorporated into carbon dots.^[78]^ In this example, the photobase acted as a mediating molecule for a proton to go from water to the catalytic site (Figure 7c), a process that took only 30 ps (as determined by time‐resolved absorption spectroscopy), with the reaction occurring at neutral pH.^[78]^ To explain the mechanism, the authors suggested that upon irradiation with light, a molecular complex is formed between the photobase and the proton donor (water), which is followed by electron transfer from the acridine photobase. Eventually, the resulting radical (AcrH⋅) can lead to the generation of H_2_ via the Heyrovsky mechanism.

The last catalytic process we will mention is related to CO_2_ capture and release, which became one of the most important processes from a sustainability perspective. In the discussed example,^[79]^ a continuous flow tube‐in‐tube system containing a suitable CO_2_ absorbent (bicarbonate solution) and HPTS photoacid was developed. The process involves an ESPT from the HPTS photoacid to bicarbonate upon the irradiation with 427 nm wavelength, resulting in the decomposition of the latter and CO_2_ gas release (Figure 7d). Consequently, the ground state recombination process can involve capturing aqueous CO_2_ to regenerate the carbonate ion. As discussed above, due to the operation mode of Brønsted photoacids, this process should not result in the acidification of the solution.

### 2.5 Switchable Proton Conductivity

All of the sections of this review until now concerned an ESPT process between Brønsted photoacids/photobases and an additional proton acceptor/donor, resulting in some chemistry with a dynamic nature. In the last section of this review, we will discuss the use of photoacids/photobases as transient sources of charge carriers (primarily a proton). Such conversion of light to electric energy is analogous to the photovoltaic effect, in which, for both cases, there is a need for charge separation after excitation, electron‐hole separation in photovoltaics or the proton‐photoacid as in here. As in photovoltaic, also for photoacids, one of the crucial steps is to prevent recombination following charge separation. As discussed, Brønsted photoacids/photobases do not result in a stable pH change due to the immediate recombination process. Thus, to use Brønsted photoacids for energy generation, there is a need to abstract the proton from the vicinity of the photoacid as fast as possible. In some of the devices discussed in this section, such separation was done by electric potential.

**Figure 8:**
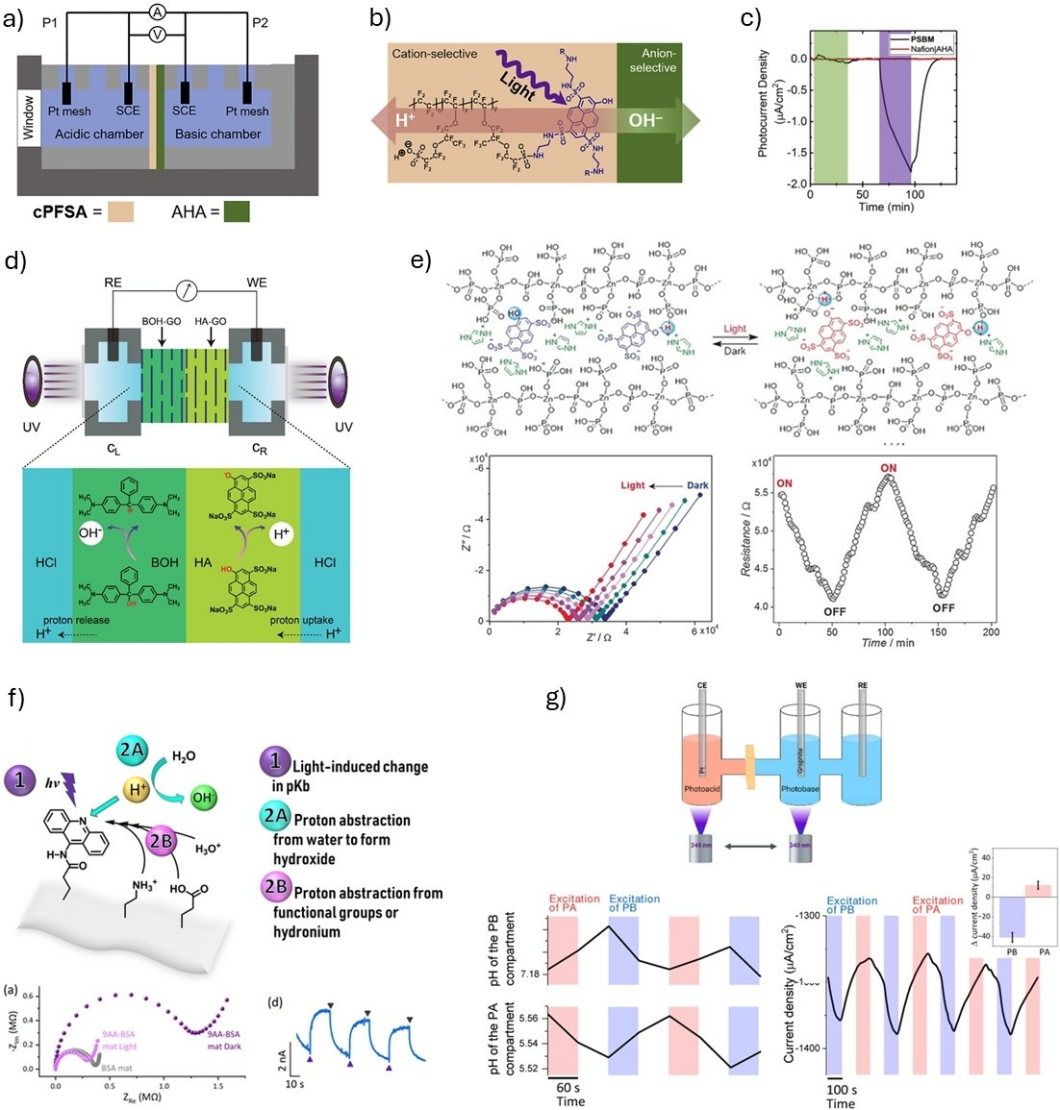
Devices and materials with switchable protonic conductivity. **a**) and **b**) Schematic representation of an electrochemical cell and the structure of the membrane, respectively. **c**) Photocurrent response of a bipolar membrane (black plot) upon the irradiation with light matching the excitation wavelength of the photoacid (purple) compared to a membrane without a photoacid (red plot) and to the excitation with a different light wavelength (green). Reproduced with permission from Ref.^[81]^ **d**) Bipolar membrane modified with a photoacid and Arrhenius photobase. Reproduced with permission from Ref.^[82]^ **e**) and **f**) Top: the mechanism of proton conduction upon the excitation of the photoacid and the photobase, bottom: plots showing the increase in conductivity upon light irradiation. Reproduced with permission from Ref.^[86]^ and Ref.,^[44]^ respectively. **g)** Top: schematic representation of an electrochemical cell, one chamber filled with a solution containing photoacid and the other with a photobase. Bottom: the effect of proton pumping is reflected in changes in pH in each chamber after light irradiation (left); the polarity of the current switches when switching from excitation of the photoacid to the photobase‐containing chamber (right). Reproduced with permission from Ref.^[43]^

One strategy to achieve charge separation by an electric field is to create an electronic asymmetric system. For instance, an electrochemical cell with a photoacid‐modified proton conducting membrane of Nafion was introduced, in which, during light irradiation, a potential difference was created across the membrane due to the ESPT process (Figure 8a and **b**). In this way, it was suggested that this process can lead to the pumping of protons from one half‐cell to another against the pH gradient.^[80]^ By replacing the membrane with a bipolar membrane consisting of the photoacid‐modified proton‐selective Nafion combined with an anion‐exchange membrane, it was shown that the ESPT process could result in an H^+^‐OH^−^ pair that can be separated using the different membrane, much like a ‘real’ photovoltaic p‐n junction diode (Figure 8c).^[81]^

Another example of a bipolar membrane is a combination of a photoacid (HPTS) modification on one side with a photobase modification (malachite green carbinol base) on the other side, albeit the photobase was not a Brønsted one but an Arrhenius OH^‐^‐releasing photobase (Figure 8d).^[82]^ Following simultaneous light irradiation (365 nm, which was shown to excite both molecules) of both membrane sides in an acidic solution, protons were released and consumed by the solution on the photoacid side of the membrane, while the released hydroxides were consumed on the photobase side. Accordingly, an asymmetric charge polarization was formed, followed by proton pumping through the membrane. Due to the use of an Arrhenius‐type photobase with a very slow recombination time, the response time and cyclability of the electrochemical cell were also very slow.

Inspired by biological proton channels, several artificial channels have been suggested, whereas a photoacid can modulate the protonic transport across them.[^83^, ^84^, ^85^] The hypothesis behind such configuration is that upon light excitation, the dissociated proton can be captured by the channel or change the surface charge of the channel, and accordingly, the protonic diffusion/conductivity across the channel can be changed. In such configurations, the photoacid can either be solvated in solution[^84^, ^85^] or chemically tethered to the surface of the channel.^[83]^ The latter configuration is adventurous as it localizes the ejected protons to the channel itself.

The driving force for the protonic charge separation can also be done electronically. In such studies, a photoacid can be introduced into a proton conductive material connected to electrodes. Accordingly, upon light irradiation and bias application, the proton that is released in the ESPT process can instantaneously become a charge carrier and, thus, increase the measured proton conductance across the material. In this way, light is used as a gating source to control the proton conductance across the material. In one of the examples, a solid‐state photosensitive proton‐conducting material was introduced, which was made of an acid‐doped coordination polymer containing a photoacid (HPTS, which was not covalently attached).^[86]^ The mechanism of photocurrent generation involved light‐triggered ESPT from the photoacid to the coordination polymer resulting in proton dissolution and mobility in the dry polymer (Figure 8e). In another example, a biopolymer was used, where either a photoacid (HPTS) or a photobase (9AA) was covalently attached to the surface of electrospun mats made by the serum albumin protein (Figure 8f for the attachment of the photobase).^[44]^ Following light‐triggering (340 nm for 9AA or 405 nm for HPTS), the conductance across the biopolymer was increased. While discussing manipulating the charge carrier concentration of an ionic conductor using a photobase, two different outcomes can be envisioned. The first is the abstraction of mobile protons by the photobase, thus decreasing the protonic conductance. The second is the abstraction of protons from water molecules, thus creating mobile hydroxides, which can be used as charge carriers and will increase measured conductance. In the study, it was shown that the second option happened.^[44]^

The last example in this section targets a tandem function involving both the proton‐conducting membrane and the electrode interface.^[43]^ In this example, it was shown that the excitation (340 nm) of either a photoacid (2 N6S) or a photobase (6AQ), i.e., the formation of either a deprotonated photoacid, protonated photobase, hydroxide or hydronium ions, next to the electrode interface can result in either cathodic or anodic photocurrent as a function of the applied overpotential. In the electrochemical setup shown in Figure 8g, while placing the electrodes in two different chambers, one containing the photoacid and the other the photobase, whereas a proton‐conductive Nafion membrane separates the chambers, there were two processes contributing to the photocurrent: electrochemical activity of ionic species formed at the electrode interface and proton pumping through the membrane. In this way, switching in the current polarity by alternating the irradiation from the photoacid containing chamber to the photobase one and vice versa was observed.

## 3 Summary and Outlook

In this review, we explored using Brønsted photoacids/photobases for light‐gating various dynamic processes and systems. We further highlighted the advantages and disadvantages of using these molecules compared to other photoswitches. In summary, Brønsted photoacids/photobases can only work if a nearby proton acceptor/donor is next to them or if the proton (or hydroxide) can be separated electronically. But, unlike other photoswitches, Brønsted photoacids/photobases do not acidify (or basicify) the solution in a stable manner, and the ESPT process is considerably faster than other proton‐releasing molecules. This mode of operation of Brønsted photoacids/photobases requires careful design and feasibility checks in a certain system to bypass the rapid proton recombination. The more recombination is avoided, the more significant the effect of the ESPT process on the system. Accordingly, our first and most important recommendation is to try to tether the photoacid/photobase the closest possible to the proton donor/acceptor, in contrast to having the photoacids/photobases soluble in solution. This is especially important considering the high absorption coefficient of some of the photoacids/photobases that will limit the light penetration depth. Photoacids and photobases tethered to biological polymers, proton‐conductive membranes or silicon surfaces were discussed in this review. This concept can be further extended to decorate the surfaces of nanoparticles to make them responsive to light stimuli. A possible direction is the development of nano‐robots for biomedical applications, capable of self‐propulsion, light‐triggered drug release, photo‐therapies and biosensing. Another promising direction is electronic devices; tethering photoacids or photobases to the surfaces of electrodes can result in light conversion/harvesting properties.

The photoacids discussed here have a p*K*_a_ of 7–9, while the photobases have a p*K*_a_ of 4–6. As discussed, the main requirement for using these molecules is that the photoacid will be protonated, and the photobase will be deprotonated upon excitation. Accordingly, the ground state p*K*_a_ of photoacids limits their use to pH values below 7, while that of photobases to pH values above 6. Hence, the race for photoacids with a very high p*K*_a_ while still having a low <1 p*K*_a_* in the excited state and for photobases with a very low ground state p*K*_a_ while still having a high >12 p*K*_a_* in the excited state is highly important for broadening the use of Brønsted photoacids/photobases.

Another important improvement for the use of photoacids and photobases is concerning their excitation wavelengths, which are usually in the UV range or going into the blue (for HPTS). Such high energy excitation can be disadvantageous for many applications, and photoacids and photobases that absorb toward the red, or even the IR, can be very attractive. Another important disadvantage of some photoacids, especially HPTS, is their relatively high photobleaching rate. Hence, this is also a property that needs to be improved. For example, a series of photoacids with emission at longer (‘redder’) wavelengths than HPTS was developed using rational design principles.^[87]^ In the latter example, p‐HBDI (4‐hydroxybenzylidene‐1,2 dimethylimidazolinone), a natural photoacid in the core of GFP, was modified to enable photoacidity outside of the protein matrix. Further functionalizations were introduced to tune the electronic properties of the ground and excited states, resulting in GFP‐like derivatives with extended emission wavelengths.

In summary, in this review, we discussed the theoretical aspects, advantages and disadvantages of photoacids and photobases and presented several applications in functional systems, including dynamic self‐assembly, self‐propulsion, photocurrent generation via ESPT, control of biological reactions, separation of biomolecules, and photocatalysis. We anticipate that the diversity of photoacids and photobases may lead to new applications.

## Conflict of Interests

The authors declare no conflict of interest.

4

## Biographical Information

*Anna Yucknovsky is a Postdoctoral Research Associate at the University of Oxford. Anna holds PhD in Chemistry, Technion. In 2023, she joined Prof. Hagan Bayley's group, where she researches nanopore chemistry*.

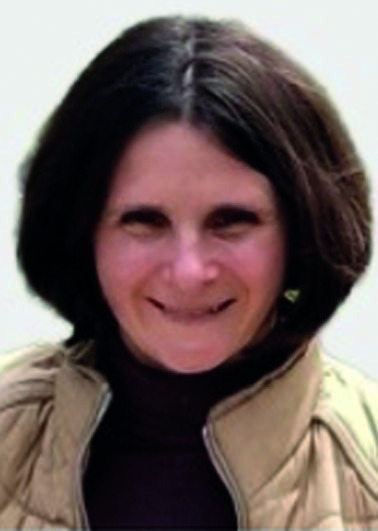

## Biographical Information

*Nadav Amdursky is a Professor of Biophysical Chemistry at the University of Sheffield. Nadav holds a PhD in Biotechnology and Electrical Engineering, Tel Aviv University, and did two postdocs on the electronic properties of biomaterials from the nano‐ to the macro‐scale at the Weizmann Institute and Imperial College. In 2016, he opened a group in the Technion, researching charge transfer, light‐gated processes, and functional biopolymers. In 2024, he opened a new group in Sheffield*.

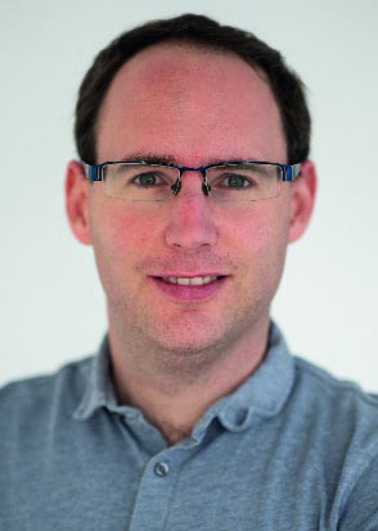

## Data Availability

Data sharing is not applicable to this article as no new data were created or analyzed in this study.
